# Measuring Organizational Culture in Ethiopia’s Primary Care System: Validation of a Practical Survey Tool for Managers

**DOI:** 10.34172/ijhpm.2022.6646

**Published:** 2022-07-11

**Authors:** Lingrui Liu, Leslie A. Curry, Kidest Nadew, Mayur M. Desai, Erika Linnander

**Affiliations:** ^1^Global Health Leadership Initiative, Yale University, New Haven, CT, USA.; ^2^Department of Health Policy and Management, Yale School of Public Health, New Haven, CT, USA.; ^3^Department of Chronic Disease Epidemiology, Yale School of Public Health, New Haven, CT, USA.

**Keywords:** Organizational Culture, Primary Care, Survey Validation, Ethiopia, Sub-Saharan Africa, Healthcare Quality

## Abstract

**Background:** Organizational culture has been widely recognized as predictive of health system performance and improved outcomes across various healthcare settings. Research on organizational culture in healthcare has been largely conducted in high-income settings, and validated scales to measure this concept in primary healthcare systems in low- and middle-income country (LMIC) settings are lacking. Our study aimed to validate a tool to measure organizational culture in the context of the Ethiopian Primary Healthcare Transformation Initiative (PTI), a collaborative of the Federal Ministry of Health (FMoH) and the Yale Global Health Leadership Initiative to strengthen primary healthcare system performance in Ethiopia.

**Methods:** Following established survey development and adaptation guidelines, we adapted a 31-item US-based organizational culture scale using (1) cognitive interviewing, (2) testing with 1176 district and zonal health officials from four regions in Ethiopia, and (3) exploratory factor analysis (EFA).

**Results:** Based on the results of cognitive interviewing, an adapted 30-item survey was piloted. The factor analyses of 1034 complete surveys (88% complete responses) identified five constructs of the scale which demonstrated strong validity and internal consistency: learning and problem solving, psychological safety, resistance to change, time for improvement, and commitment to the organization. Of the 30 a priori items, 26 items loaded well on the five constructs (loading values 0.40-0.86), and 4 items failed to load. Cronbach alpha coefficients were 0.86 for the scale as a whole and ranged from 0.65 to 0.90 for the subscales. The five-factor solution accounted for 62% of total variance in culture scores across respondents.

**Conclusion:** Through validation and factor analyses, we generated a 26-item scale for measuring organizational culture in public primary healthcare systems in LMIC settings. This validated tool can be useful for managers, implementers, policy-makers, and researchers to assess and improve organizational culture in support of improved primary healthcare system performance.

## Background

**Key Messages**

** Implications for policy makers**

- Our study adapted and validated a 26-item scale for measuring organizational culture in Ethiopia’s primary healthcare system, which provides a broader measure of organizational culture compared to relatively circumscribed measures of safety climate that have been commonly used in low- and middle-income country (LMIC) settings.
- The resulting scale was perceived by primary healthcare professionals at the district and woreda level to be feasible to implement and valuable as a practical tool for measuring organizational culture to improve primary healthcare performance.
- Our results reinforce the importance of fostering organizational learning as a cornerstone of effective organizational culture.

** Implications for the public**

Organizational culture has been associated with healthcare system performance and improved patient outcomes in various healthcare settings in high-income countries (HICs). Despite a growing recognition of this concept in healthcare systems in low- and middle-income countries (LMICs), considerable literature has focused on the value of safety climate in hospitals in LMICs, which are more narrowly circumscribed within the broader measures of organizational culture. Yet, there has been no rigorously validated organization culture scale tool in primary healthcare systems in LMICs for healthcare professional and other stakeholders to feasibly implement and use. Our study adapted and validated the organizational culture measures perceived by healthcare professionals at the district and woreda levels in Ethiopia’s primary care system. The resulting tool, a 26-item scale, provides managers, implementers, policy-makers, and other stakeholders a useful tool to determine and improve organizational culture for improved primary healthcare system performance.

Organizational culture is defined as a set of shared social constructs about “the way we do things around here” that are shaped and accumulated as an organization’s members interact with each other and with the external environment.^1^ An organization’s culture is predictive of health system performance and improved patient outcomes across a number of health conditions and healthcare settings.^2-5^ In high-income country (HIC) settings, organizational culture has been positively associated with a range of hospital system performance measures and complex patient outcomes including mortality rates, falls and infections, readmission rates,^6,7^ adverse events, medication errors, and patient satisfaction.^3,8-10^ In the vast majority of the studies in HIC settings, the findings were based on observational research conducted in hospitals, with a small but growing body of research in primary healthcare,^11^ senior care, and mental healthcare.^8^ In low- and middle-income country (LMIC) settings, a growing body of research on organizational culture has focused on the association between hospital safety culture and patient outcomes.^12-16^ Further, a systematic review of qualitative and ethnographic studies of public health systems in LMICs demonstrated the important role of organizational culture in the success of LMIC health sector reforms, positing that health sector redesign^17^ is insufficient to achieve global health targets, and that intangible aspects of inner settings (ie, organizational culture) drive the functions, operations, and relationships within each health systems.^17^

 Despite this growing body of evidence supporting the role of organizational culture in efforts to strengthen health systems in LMIC settings, policy-makers and researchers lack tools to meaningfully measure organizational culture.^17^ Studies of organizational culture in high-income settings rely on validated measurement tools.^11,18-21^ However, the extent to which these measures are valid and reliable across country contexts (translating from HIC to LMIC) or care settings (translating from hospital-based to primary care contexts) is unknown. We found a number of examples of LMIC studies in which a tool was directly imported,^13,14,22,23^ one in which an existing scale (the Organizational Culture Assessment Instrument) was adapted and validated in an LMIC hospital setting,^24^ and no studies offering LMIC-validated scales for measuring organizational culture in the context of primary healthcare. Therefore, we set out to adapt a validated tool to measure organizational culture in the context of Ethiopia’s primary healthcare system. We describe the adaptation process and present the psychometric properties of the resulting scale, developed and tested at the district level across four regions in the context of Ethiopia’s Primary Healthcare Transformation Initiative (PTI).^25^ The description of the adaptation is expected to be useful to researchers and practitioners seeking to adapt existing measures of organizational culture to their unique contexts. In addition, the resulting tool may be useful for managers in LMIC settings seeking to assess their own organizational culture, as well as implementers, policy-makers, and researchers seeking to evaluate efforts to proactively improve organizational culture in support of improved healthcare system performance.

## Methods

###  Setting

 We conducted this study in Ethiopia, a lower-income country in which 84% of the population lives in rural areas.^26^ Through rapid expansion of the primary healthcare system, Ethiopia has achieved most of the health Millennium Development Goals, including significant reductions in preventable childhood and maternal mortality, and compelling decreases in communicable diseases.^27,28^ In Ethiopia’s primary healthcare system, the zonal health department is responsible for primary healthcare services across 15-20 woredas (districts), each of which, on average, includes 4-5 health centers and a health extension program. Primary care activities are organized and integrated at the district level, while the zonal health department plays important roles in performance management, resource allocation, and cross-sectoral integration. Through the Health Sector Transformation Plan and accompanying Woreda Transformation Plan, the government has articulated an ambitious vision for primary healthcare system reform. Based on prior systematic review^17^ of health sector reform in LMIC settings, implementation of Ethiopia’s plans is likely to require investment in fostering an organizational culture that supports improved care delivery.

###  Study Sample

 The sample for this study were district and zonal health officers that participated in the in the Ethiopian PTI, which has been described elsewhere.^25,29^ Briefly, PTI, a collaboration between the Ethiopia Federal Ministry of Health (FMoH) and the Yale Global Health Leadership Initiative, was conducted in four regions: Amhara, Oromia, Southern Nations Nationalities and Peoples (SNNP), and Tigray. PTI aimed to build a culture of performance management and accountability, and thereby improve the effectiveness of districts in leading the ambitious set of primary healthcare reforms envisioned by the FMoH in its Health Sector Transformation Plan 2015-2020. PTI Phase I (2015-2017), focused on 36 woreda health offices. Phase II (2017-2019) focused on 17 zonal health offices (across Amhara, Oromia, and SNNP) and 2 clusters (in Tigray, where the zonal structure is not used), which covered 315 woredas and 1617 health centers, and served a population of 47 million. In both phases, sites were selected for intervention through collaboration with regional health bureaus based on criteria including receptivity to the intervention and size of population. Zonal and district health officials in each PTI site used the organizational culture tool described herein as part of their efforts to understand and improve ways of working.

###  Validated Scale

 We began with an existing scale for measuring organizational culture that had been designed and validated in the context of a US-based leadership development initiative called Leadership Saves Lives (LSL).^9,19^ Although the exiting scale was validated in the context of US hospitals (not primary care), recent systematic review to identify validated measures of organizational culture used in primary care within recent years (2008–2019) found wide variability and major limitations in both conceptual design and psychometric quality.^11^ In contrast, LSL and PTI relied upon similar theories of change; both were leadership development interventions designed to foster creative problem solving, interdisciplinary and interorganizational collaboration, and progress toward improved performance. The 31-item scale validated in the context of LSL measured five dimensions of organizational culture: (1) learning environment, (2) psychological safety, (3) commitment to the organization, (4) senior management support, and (5) time for improvement efforts, all of which were hypothesized to be relevant in contexts beyond the hospital setting.

###  Adaptation

 To adapt the tool to the Ethiopian context, we used the World Health Organization (WHO) guidelines on the process of translation and adaptation of instruments to guide our iterative approach to adaptation.^30^ We updated the wording of each item to replace references to cardiovascular care in hospitals with reference to primary care in woredas (districts). We then conducted cognitive interviews^31^ with a senior regional manager from each of the 4 intervention regions. Senior regional managers are members of the PTI team that have professional backgrounds in primary care, come from the regions in which PTI is working, and have a deep understanding of the PTI approach. Based on results of the cognitive interviews, the survey tool was modified as follows:

- One question from the factor “Commitment to the organization” (I would be very happy to spend the rest of my career at this hospital) was removed because of perceived lack of relevance to the predominantly government-run primary healthcare system.
- The phrase “In this work environment” was dropped from each item and replaced with brief instructions preceding the scale: “Please answer the following questions based on your experiences supporting primary healthcare in your woreda.”
- Minor adjustments to phrasing were made for clarity; the roots of each item were unchanged.

The resulting survey was translated and back-translated into three local languages (Amharic, Oromifa, and Tigrinya). A pilot testing using local language surveys was conducted in the 243 health professionals in PTI Phase I districts to assess feasibility of implementation, and to elicit informal feedback on the usefulness and relevance of the tool to managers in the primary healthcare system. PTI management mentors based in each Phase I district reported feeling able to use the results to promote reflection among participants on local organizational culture in primary care, with an eye toward improvement in ways of working and primary care system performance improvement. Figure presents the full adaption process.

**Figure:**
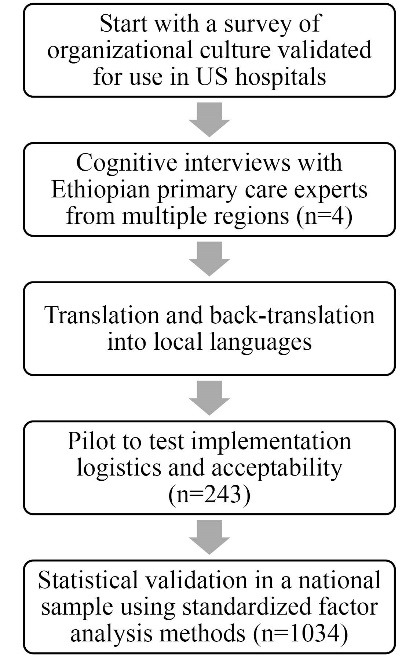
The Adaptation and Validation Process.

###  Validation and Factor Analysis

 The survey response choices consisted of a 5-point Likert scale with options for strongly agree (5), agree (4), neutral (3), disagree (2), and strongly disagree (1). As recommended for enhancing psychometric rigor,^32^ the survey included a mix of both positively and negatively phased questions. To be comparable with the validation results of the US LSL organizational culture survey, the scoring of responses was inverted as needed during analysis so that lower scores would consistently reflect more desirable culture.

 We estimated the mean scores and standard deviation (SD) for each item and for overall culture. To assess construct validity, we used exploratory factor analysis (EFA)^33^ to identify the factors that explain the common variance (the amount of variance that is shared among a set of items) among survey responses. We confirmed the sampling adequacy for factor analysis using the Kaiser-Meyer-Olkin test and that the items were correlated (ie, did not result in an identity matrix) using the Bartlett’s test of sphericity.

 We performed EFA using promax oblique rotation to consider potential correlations between the organizational culture factors. We used a threshold of 0.40 for factor loadings.^34^ To determine the optimal number of factors to retain in the EFA, we applied Kaiser’s rule of retaining factors whose eigenvalues are greater than 1,^35^ based on the assumption that a factor with eigenvalue less than 1 explains less variance than a single original variable, which is not psychometrically reasonable.^36^ For the items which had cross-loadings, we conducted different rotation methods to eliminate cross-loadings and simplify the structure.

 We assessed the reliability of the final factors using Cronbach alpha.^37^ Consistent with widely used guidelines, a Cronbach alpha score of ≥0.65 was considered of acceptable internal consistency.^38^ In addition to examining factor reliability, we also computed the Cronbach alpha score of the 30-item organizational culture scale as a whole. To test goodness of fit, we also performed confirmatory factor analysis on the full sample.^39^ The statistical analyses in this study were performed using Stata SE15.1 (StataCorp, Texas).

## Results

###  Sample

 The resulting sample included 1,176 respondents (100% response rate), 1034 (87.9%) of which had responses for each item: 176 (94.1%) respondents from Amhara, 377 (92.2%) from Oromia, 242 (91.7%) from SNNP, and 239 (75.5%) from Tigray.

Table 1 presents the mean score for each item as well as for the overall 30-item scale. After the negatively phrased questions were reversely coded, the average score across all items ranged from 2.3 to 3.6, and for the overall scale, the average score was 2.8 (SD: 0.5) on the 5-point Likert scale where lower scores are interpreted as more positive ratings of organizational culture.

**Table 1:** Distribution of Responses to Each Item (N = 1034)

**Item**^a^	**Statement**	**Mean**^b^	**SD**
Q1	We are encouraged by management to come up with new (innovative) ways to solve problems related to primary healthcare.	2.7	1.2
Q2	There is good coordination among the different primary healthcare facilities and woreda health office in the woreda.	2.8	1.2
Q3	We have created mechanisms to hold each other accountable for high quality care.	2.9	1.2
Q4	We rely on data to guide our improvement processes.	2.6	1.2
Q5	We have frequent interactions with outside organizations to acquire new knowledge and experience on how to improve primary healthcare.	3.0	1.2
Q6	This work environment encourages people to be interested in better ways of doing things.	2.8	1.2
Q7R	In this work environment, people often resist new approaches.	2.7	1.2
Q8	In this work environment, people value new ideas.	2.6	1.1
Q9	Despite the workload, people in this work environment find time to review how the work is going.	2.6	1.1
Q10	In this work environment, someone makes sure that we stop to reflect on the team’s work process.	2.7	1.2
Q11R	If you make a mistake in this work environment, you will pay a price for it.	3.4	1.2
Q12	People in this work environment are encouraged to bring up problems and tough issues for discussion.	2.6	1.2
Q13R	In this work environment, there are people who deliberately act to undermine my efforts.	2.6	1.2
Q14R	It is difficult to ask others in this work environment for help.	2.4	1.2
Q15	In this work environment, people’s unique skills and attributes are valued and utilized.	3.1	1.3
Q16	People in this work environment express their view freely and opposing views are welcome.	2.8	1.2
Q17	The senior management has set improving primary healthcare as a priority and is working on it.	2.7	1.2
Q18	The senior management believes that current practices for primary healthcare should be improved.	2.4	1.2
Q19	The senior management has encouraged changes in practices to improve primary healthcare.	2.5	1.2
Q20	The necessary financial resources for personnel and equipment are provided for primary healthcare.	3.3	1.2
Q21	I enjoy discussing my organization with people outside of it.	3.1	1.3
Q22R	I think I could easily become as attached, if i move to another organization as I am to this one.	3.6	1.1
Q23R	I do not feel like ‘part of the family’ in this organization.	2.6	1.4
Q24R	I do not feel ‘emotionally attached’ to this organization.	2.7	1.3
Q25	This organization has a great deal of personal meaning to me.	2.7	1.3
Q26R	I do not feel a strong sense of belonging to this organization.	2.3	1.3
Q27R	In this work environment, people caring for patients are overly stressed.	3.1	1.3
Q28R	In this work environment, the time pressure gets in the way of doing a good job.	2.9	1.3
Q29R	In this work environment, people are too busy to invest time in service improvement.	2.6	1.2
Q30R	There is no time to review and revise work process in this work environment.	2.5	1.3
Overall score		2.8	0.5

Abbreviation: SD, standard deviation. ^a^Item represents the ascending order (from the 1st to the 30th item) of the survey items which was administered. ^b^1 = strongly agree, 2 = agree, 3 = neutral, 4 = disagree, 5 = strongly disagree. The lower scores indicate the more desirable culture perception after negatively phrased questions (denoted with an R) were reverse coded.

###  Factor Analysis 

 The Kaiser-Meyer-Olkin value of our sample size was 0.90 and Bartlett’s test of sphericity was significant with a *P* value of <.001. The results indicate that the data set is adequately sampled, and that factor analysis of the data is appropriate. Therefore, we proceeded with EFA and all 30 items were included in the EFA. Based on eigenvalues of the correlation matrix, we also proceeded the scree plot test^40^ and examined that the sharp drop-off in the scree plot occurred after 5 factors. Therefore, five factors were retained through the factor analysis, where 26 items loaded on one of these five factors with loadings above 0.40. Four items failed to load on the factors. Three of these four items (Q21 “I enjoy discussing my organization with people outside of it,” Q22R “I think I could easily become as attached to another organization as I am to this one,” Q25 “This organization has a great deal of personal meaning to me”) were from the factor “Commitment to the organization.” Table 2 shows the factor analysis loadings on the five constructs (factors) which were identified: (1) learning and problem solving, (2) psychological safety, (3) resistance to Change, (4) time for improvement, (5) commitment to the organization. With the promax oblique rotation, items loaded significantly only on one factor, and no cross-loadings exist. The 5 factors had loading values between 0.40 and 0.86. As the EFA guideline recommended, we re-estimated the model with 6 factors and 7 factors, respectively, retained in the solution. In the model re-estimations, items did not load meaningfully on the sixth or seventh factor. The visualized presentation of scree plot suggested that the 5 factors were most appropriate. The 5-factor solution accounted for 62% of total variance, which meets the recommended threshold that factors should explain 50% of total variance.^41^

**Table 2:** Factor Analysis Loadings (N = 1034)^a^

		**Learning and Problem Solving**	**Psychological Safety**	**Resistance to Change **	**Time for Improvement**	**Commitment to the Organization**
**Alpha Scores **(an overall Chronbach alpha score of the 30-item as a whole: 0.86)		0.90	0.65	0.65	0.74	0.74
**Statement**	**Item**^b^					
We are encouraged to come up with new ways to solve problems.	Q1	0.75				
Good coordination among the different facilities.	Q2	0.63				
We created mechanisms to hold each other accountable.	Q3	0.56				
We rely on data to guide improvement process.	Q4	0.53				
We have frequent interactions with outside organizations to acquire new knowledge and experience on how to improve primary healthcare.	Q5	0.56				
This work environment encourages people to be interested in better ways of doing things.	Q6	0.65				
In this work environment, people’s unique skills and attributes are valued and utilized.	Q15	0.47				
People in this work environment express their view freely and opposing views are welcome.	Q16	0.67				
The senior management has set improving primary healthcare as a priority and is working on it.	Q17	0.81				
The senior management believes that current practices for primary healthcare should be improved.	Q18	0.79				
The senior management has encouraged changes in practices to improve primary healthcare.	Q19	0.86				
The necessary financial resources for personnel and equipment are provided for primary healthcare.	Q20	0.67				
If you make a mistake in this work environment, you will pay a price for it.	Q11R		-0.58			
People in this work environment are encouraged to bring up problems and tough issues for discussion.	Q12		0.54			
In this work environment, people value new ideas.	Q8		0.78			
In this work environment, someone makes sure that we stop to reflect on the team’s work process.	Q10		0.52			
In this work environment, people often resist new approaches.	Q7R			0.69		
In this work environment, there are people who deliberately act to undermine my efforts.	Q13R			0.74		
It is difficult to ask others in this work environment for help.	Q14R			0.40		
In this work environment, people caring for patients are overly stressed.	Q27				0.67	
In this work environment, the time pressure gets in the way of doing a good job.	Q28				0.72	
In this work environment, people are too busy to invest time in service improvement.	Q29				0.82	
There is no time to review and revise work process in this work environment.	Q30				0.75	
I do not feel like ‘part of the family’ in this organization.	Q23R					0.83
I do not feel ‘emotionally attached’ to this organization.	Q24R					0.84
I do not feel a strong sense of belonging to this organization.	Q26R					0.59
Despite the workload, people in this work environment find time to review how the work is going.	Q9			Failed to load		
I enjoy discussing my organization with people outside of it.	Q21			Failed to load		
I think I could easily become as attached to another organization as I am to this one.	Q22R			Failed to load		
This organization has a great deal of personal meaning to me.	Q25			Failed to load		

^a^The factor analysis used 0.4 as the threshold for factor loading. Only factor loadings >0.40 are presented in the table. ^b^Item represents the ascending order (from the 1st to the 30th item) in which the survey items were administered.

 To assess internal consistency, we calculated Cronbach alpha for each of the five factors and for the 30-item scale as a whole, as well as for the 26-item final scale excluding the four failed-to-load items. Factor 1 (learning and problem solving), factor 4 (time for improvement), and factor 5 (commitment to the organization) had strong internal consistencies (0.90, 0.74, and 0.74, respectively), all substantially exceeded the threshold of acceptability.^32^ Factor 2 (psychological safety) and factor 3 (resistance to change) had moderate consistencies (0.65 and 0.65). The Cronbach alpha for the 30-item scale as a whole and for the 26-item final scale excluding the four failed-to-load items was 0.86 and 0.84, respectively, indicating a strong internal consistency. To test the goodness of fit of the model, we conducted confirmatory factor analysis using the structural equation modeling. The goodness-of-fit indices were above the thresholds of acceptability (see Supplementary file 1, Table S1 for detail fits of statistics).^39^

## Discussion

 We successfully adapted and validated a 26-item scale for measuring organizational culture in Ethiopia’s primary healthcare system. Like the original scale, the adapted scale included 5 domains, each with moderate to strong levels of internal consistency. As highlighted through systematic reviews in HIC settings, there is no consensus on which domains or subconstructs comprise the construct of organizational culture.^11,42,43^ However, there is strong theoretical grounding for each of the resulting domains: learning and problem solving,^44-46^ psychological safety,^47^ resistance to change, time for improvement,^48-50^ commitment to the organization. Notably, the resulting scale provides a broader measure of organizational culture as compared to more narrowly circumscribed measures of safety culture that are becoming increasingly common in LMIC settings.

 The resulting tool was perceived by primary healthcare professionals at the district and woreda level to be feasible to implement and valuable as a practical tool for measuring organizational culture in the context of efforts to improve primary healthcare performance. Some professionals participating in the piloting of the tool saw potential to use it as a jumping-off point for facility- and district-level reflection and action planning to improve local organizational culture. Others believed the measure could eventually be included in the emerging national systems for primary care performance monitoring and improvement, similar to Ethiopia’s existing hospital-level performance monitoring systems^51^ and improvement collaboratives.^52^ Comparisons with the a priori tool demonstrate ways in which systematic adaptation and validation in new country contexts and healthcare setting can be helpful. First, although affective commitment to the organization, or a feeling of connection and wanting to stay with the organization,^53^ is predictive of organizational performance across sectors and settings,^54^ our results highlight that this domain requires careful adaptation to be made relevant for government healthcare workers. One item from this a priori domain was removed during cognitive interviewing, and three others failed to load during factor analysis. This is consistent with prior literature^55^ suggesting that the association between overall culture, affective commitment, and performance is attenuated in highly bureaucratic public organizations. Second, our results demonstrate that the use of both positively- and negatively-phrased items requires careful attention when working across linguistic contexts. For example, the a priori domain of psychological safety factored into two unique domains in the Ethiopian context (psychological safety and resistance to change), largely along the lines of positively- vs negatively-phrased items. Finally, our results reinforce the importance of fostering organizational learning as a cornerstone of effective organizational culture. Of the 30 items administered, 12 factored into the learning and problem solving domain, representing diverse constructs such as senior management support, access to resources, and use of data for accountability.^56^

 Our results should be interpreted in light of several limitations. First, we have not yet evaluated the extent to which the resulting survey is predictive of primary healthcare system performance. However, the importance of organizational culture in shaping performance is well established,^8,10,17,57^ and a large number of staff using the tool in Ethiopia reported that the measures were relevant and helpful in their performance improvement work. Second, we have not yet assessed sensitivity of the scale to detect change over time. However, in the context of LSL, the scale was effective at detecting changes in organizational culture over a two-year time period,^9^ even with relatively small sample sizes (12-20 respondents per hospital), similar to our target participant groups in Ethiopia. Third, responses may have been influenced by social desirability bias.^58^ However, the likelihood of this was reduced by the fact that survey responses were anonymous, and showed enough variation to generate domains similar to those hypothesized based on prior work, strengthening confidence in our results.

## Conclusion

 Measurement of organizational culture is an important input for efforts to strengthen primary healthcare system performance in LMIC settings. However, systematic adaptation of existing tools to local context is essential. Through validation and factor analysis, we generated a 26-item scale that was meaningful, actionable, and feasible district- and zonal-level managers to administer. The resulting scale is expected to be useful to health professionals in LMIC settings seeking to measure and improve organizational culture, and to researchers seeking to understand and compare organizational culture change efforts across contexts.

## Acknowledgements

 The authors gratefully acknowledge the respondents from each participating woreda for their commitment to excellence; the Regional Health Bureaus and FMoH for their vision and support; and the PTI Technical Advisors for Management Systems and Senior Regional Managers for their service as changemakers in primary care. Preliminary results associated with the piloting of this tool were presented at the IHI Africa Forum 2018, where valuable comments were received from conference participants. The authors also thank Dr. Emily Cherlin for her valuable inputs to the development of this paper.

## Ethical issues

 The study, which included no personal identifiers, was deemed to be exempt from continuing review by the Institutional Review Board at Yale University. The Federal Republic of Ethiopia Ministry of Health approved the project by submitting a Letter of Support. The need for ethics approval was deemed unnecessary by the Federal Republic of Ethiopia Ministry of Health according to Ethiopian regulations because this study did not collect personal identifiers or individual-level health information. This study was performed in accordance with the Declaration of Helsinki as part of the Yale Global Health Leadership Initiative’s delivery of the Primary Healthcare Transformation Initiative (PTI). Completion of the survey was voluntary and anonymous, and respondents provided informed consent to participate.

## Competing interests

 Authors declare that they have no competing interests.

## Authors’ contributions

 Conceptualization: LL, KN, LC, and EL. Data curation: LL, KN. Funding acquisition: EL. Methodology: LL, LC, MD, and EL. Project administration: KN, EL. Supervision: LC, EL. Visualization: LL. Writing–original draft: LL, EL. Writing–review: LL, LC, KN, MD, and EL.

## Funding

 This study was conducted in the context of the Primary Healthcare Transformation Initiative (PTI), which was funded by a grant to Yale University from the Bill & Melinda Gates Foundation.

## Supplementary files

Supplementary file 1 contains Table S1.
