# Supplementary material for: Measuring Organizational Culture in Ethiopia’s Primary Care System: Validation of a Practical Survey Tool for Managers
Source: Int J Health Policy Manag. 2022 Jul 11;11(12):3071–8. doi: 10.34172/ijhpm.2022.6646 (PMC10105185; doi:10.34172/ijhpm.2022.6646)
Supplement: Supplementary file 1 — contains Table S1. [file ijhpm-11-3071-s001.pdf]

Article title: Measuring Organizational Culture in Ethiopia's Primary Care System: Validation of a Practical Survey Tool for Managers

Journal name: International Journal of Health Policy and Management (IJHPM)

Authors' information: Lingrui Liu[1,2]*, Leslie A. Curry[2], Kidest Nadew[1,2], Mayur M. Desai[1,3], Erika Linnander[1,2]

[1]Global Health Leadership Initiative, Yale University, New Haven, CT, USA.

[2]Department of Health Policy and Management, Yale School of Public Health, New Haven, CT, USA.

[3]Department of Chronic Disease Epidemiology, Yale School of Public Health, New Haven, CT, USA.

(Corresponding author: lingrui.liu@yale.edu)

Supplementary file 1

Table 1. Goodness of Fit Indices.

**Likelihood Ratio**	
Chi2_ms (model vs. saturated)<br>p> chi2	1809.49<br><0.001
Chi2_bs (baseline vs. saturated)<br>p> chi2	10094.322<br><0.001
**Information Criteria**	
Akaike (AIC, Akaike's Information Criterion)	82015.854
Bayesian (BIC, Swartz's Bayesian Information Criterion)	82470.444
**RMSEA (Root Mean Square Error of Approximation)**	
Estimate	0.068
90 Percent C.I. (Confidence Interval)	0.065- 0.070
Probability RMSEA <= .05	<0.001
**Baseline comparison**	
CFI (Comparative Fit Index)	0.90
TLI (Tucker-Lewis Index)	0.90
**Size of residuals**	
SRMR (Standardized Root Mean Square Residual)	0.06
CD (Coefficient of determination)	0.99
